# Inadvertently transected left superior pulmonary vein during thoracoscopic left lower lobectomy

**DOI:** 10.1186/s13019-016-0469-0

**Published:** 2016-05-26

**Authors:** Yangki Seok, Eungbae Lee

**Affiliations:** Department of Thoracic and Cardiovascular Surgery, Kyungpook National University Medical Center, 807 Hogukno, Buk-gu, Daegu, 41404 Republic of Korea

**Keywords:** Anatomical anomaly, Pulmonary vein, Lung cancer surgery

## Abstract

**Background:**

There are several anatomical variations of the pulmonary vein which can cause serious complications in pulmonary lobectomy.

**Case presentation:**

We inadvertently divided the left superior pulmonary vein during thoracoscopic left lower lobectomy in a lung cancer patient. Retrospective review of the preoperative computed tomography showed extra-pericardial common trunk of the left pulmonary venous system. Left superior pulmonary vein was reimplanted into stump of divided common trunk via thoracotomy.

**Conclusions:**

Awareness of vascular anomalies will help thoracic surgeons to prevent potential morbidity and mortality from complications.

## Background

Pulmonary vascular variations are significant in thoracic surgery as such vascular anomalies can present a number of surgical complications [1]. Dividing a pulmonary vein that should be preserved can lead to potentially life-threatening complications [2]. In this report we describe a case where we inadvertently divided the left superior pulmonary vein (LSPV) during thoracoscopic left lower lobectomy in a lung cancer patient.

## Case presentation

**Fig. 1:**
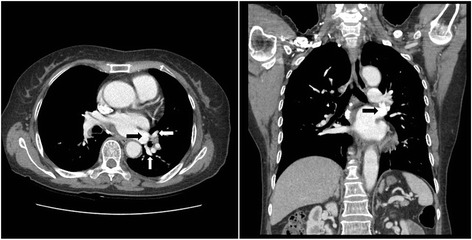
Preoperative chest computed tomography showed that superior and inferior pulmonary veins (*white arrow*) had formed a extrapericardial common trunk (*black arrow*)

The chest computed tomography (CT) of a 74-year-old female showed a lobulated cancer in the lateral basal segment of the left lower lobe measuring 1.5 cm in diameter, for which the patient underwent thoracoscopic left lower lobectomy. During this operation, the pulmonary vein was initially divided using a 2.5-mm stapling device (Echelon Flex 60 Endopath Stapler, Ethicon Endo-Surgery, LLC, Guaynabo, Puerto Rico 00969 USA). Next, the common basal and the superior segmental arteries were divided simultaneously by employing the same device, then the left lower lobar bronchus was divided using a 4.1-mm stapling device (Echelon Flex 60 Endopath Stapler). During post-lobectomy exploration, the LSPV was not be visualized in the expected location. Retrospective review of the preoperative chest CT showed anatomical variation in the pulmonary venous system. In this case, the superior and inferior pulmonary veins had formed a common trunk outside the pericardium and drained into the left atrium (Fig. 1). After heparin injection, all staples on the LSPV and an half of the staples on the common trunk were removed under intra-pericardial partial clamping of left atrium (LA) via thoracotomy. The patient became bradycardic during first attempt of partial clamping of the LA. It was difficult to clamp the LA without causing bradycardia, and the operation only proceeded after the vital signs were stable for at least 5 min after clamping. The LSPV was reimplanted into the stump of the divided common trunk using continuous 4–0 Prolene sutures. The LSPV was reimplanted in the location of the left inferior pulmonary vein in the stump of the divided common trunk, rather than its original site. This was because the length of the original site of the LSPV was shortened due to the stapling direction at the time of pulmonary vein division, and the length for reimplantation could not be approximated. The patient recovered well from the procedure and was transferred to the general ward, and was discharged 6 days after the operation without any complications.

## Discussion

Pulmonary vascular anomalies are significant in thoracic surgery [1]. Thoracic surgeons tend to focus more on variations of the pulmonary arteries than the pulmonary veins during preoperative evaluation because the majority of severe intra-operative complications during pulmonary lobectomy are related to the injury of major pulmonary arteries [3]. However, pulmonary venous anatomical variations are more common than those of pulmonary arterial branches [4]. Ligation of a pulmonary vein that should be preserved can lead to severe pulmonary edema, which may cause potentially life-threatening complications such as infection and respiratory distress [2]. Disruption of the pulmonary vein can also lead to complicated and longer procedures [5].

In consideration of the drainage pattern of the left pulmonary vein, the incidence of the common trunk forming one ostium in the LA is 14 % [6]. This can be divided into two types: the first type where the common trunk vein is less than 1 cm long, and the other where it is more than 1 cm long that drains into the LA. The incidence of having a common trunk that is longer than 1 cm is 3.5 % [6]. In this case, the superior and inferior pulmonary veins formed an extra-pericardial common trunk which was longer than 1 cm. The LSPV was reimplanted after lower lobectomy, because we had not confirmed the exact vascular anatomy and divided the common trunk consisting of the superior vein and the inferior vein, mistaking it as the inferior pulmonary vein. This is because of the surgeons’ tendency to only visualize the local surgical field rather than checking the general vascular anatomy around the operation site in thoracoscopic surgery. Retrospective review of the preoperative CT showed that the superior and inferior pulmonary veins joined to form the common trunk, which suggests that preoperative diagnosis of this variation is quite possible.

## Conclusion

Awareness of vascular anomalies is very important in excising the pulmonary lobe for lung cancer. Especially closer attention is required in thoracoscopic procedures as the surgical view is more limited than in a thoracotomy. Keeping such vascular anomalies in mind will help thoracic surgeons to prevent potential morbidity and mortality from complications.

## Abbreviations

CT: computed tomography
LA: left atrium
LSPV: left superior pulmonary vein
